# Assessment of the Effect of Selenium Supplementation on Production of Selected Cytokines in Women with Hashimoto’s Thyroiditis

**DOI:** 10.3390/nu14142869

**Published:** 2022-07-13

**Authors:** Jadwiga Kryczyk-Kozioł, Ewelina Prochownik, Anna Błażewska-Gruszczyk, Marian Słowiaczek, Qian Sun, Lutz Schomburg, Ewa Ochab, Mirosław Bartyzel, Paweł Zagrodzki

**Affiliations:** 1 Department of Food Chemistry and Nutrition, Jagiellonian University Medical College, Medyczna 9, 30-688 Kraków, Poland; jadwiga.kryczyk@uj.edu.pl (J.K.-K.); ewelina.gajdzik@uj.edu.pl (E.P.); 2 Medical Center DIAGNOZA, Zaczarowane Koło 5, 30-087 Kraków, Poland; annablazewskagruszczyk@gmail.com (A.B.-G.); marian.slowiaczek@gmail.com (M.S.); 3 Institute for Experimental Endocrinology, Charité-Universitätsmedizin Berlin, Augustenburger Platz 1, 13353 Berlin, Germany; qian.sun@charite.de (Q.S.); lutz.schomburg@charite.de (L.S.); 4 Henryk Niewodniczański Institute of Nuclear Physics, Polish Academy of Sciences, Radzikowskiego 152, 31-342 Kraków, Poland; miroslaw.bartyzel@ifj.edu.pl

**Keywords:** selenium, sodium selenite (IV), Hashimoto’s thyroiditis, thyroid disease, autoimmune process, immune system, cytokines, T cells, PLS model

## Abstract

The impact of selenium on the course of Hashimoto’s thyroiditis (HT) was mainly assessed by monitoring the titer of antithyroid autoantibodies in most of the studies conducted hitherto. On the other hand, the imbalance in activity of T cells such as Th1, Th2, Th17, and Treg may be relevant in the pathogenesis of this disease. Hence, the assessment of changes in the secretion of cytokines by these cells during selenium supplementation in patients with HT seems to be an important issue and was the main goal of this study. A further aim was to search for correlations among these cytokines, as well as markers of thyroid function, selenium/iodine status in the body, and other biochemical parameters. The group of 29 women with newly diagnosed Hashimoto’s thyroiditis was supplemented with selenium in a dose of 100 µg/day for 6 months. Immunological parameters: interferon γ, tumor necrosis factor α, chemokine CXCL10, interleukin 4, interleukin 1β, interleukin 17, transforming growth factor β, and C-reactive protein, as well as selenium status parameters were determined in serum twice, i.e., before and after supplementation. Selenium supplementation was associated with a change in the production of two cytokines: interferon γ and interleukin 1β, for which a decrease and an increase in concentration were observed, respectively. The partial least squares (PLS) model revealed the presence of many relevant correlations among analyzed parameters. The stage of HT development, degree of thyroid dysfunction, and selenium supplementation of diet are interdependent factors which shape the profile of some cytokines secreted by cells participating in the autoimmunity process.

## 1. Introduction

Hashimoto’s thyroiditis (HT) is one of the most common autoimmune diseases [1,2]. From an immunological point of view, HT is characterized by the development of immune response against thyroid self-antigens, such as thyroid peroxidase or thyroglobulin [3,4]. However, the knowledge of mechanisms underlying the disease is still incomplete. The imbalance between two subpopulations of T helper cells, i.e., Th1 and Th2 in favor of Th1, was observed in some studies on the pathogenesis of HT [5,6]. In recent years, attention has been also paid to Th17 lymphocytes which, like Th1, are characterized by pro-inflammatory properties [7,8]. It is supposed that in the initial stages of HT, a Th17-dependent immune response is dominant, whereas increased activity of Th1 cells occurs in its later stages or in the exacerbation of disease [9]. Such variation in the dominance of particular subpopulations of the Th cell during the development of HT may indicate the plasticity of the differentiation process of these lymphocytes [10]. The regulatory T cells (Treg) may also play an important role in the pathogenesis of this disease [11,12,13].

The role of selenium in autoimmune processes is not fully understood; nevertheless, there are many indications of immunoregulatory properties of this microelement, relevant in the context of Hashimoto’s thyroiditis. For example, it participates in the regulation of a number of regulatory T cells, which are involved in maintaining a peripheral tolerance. In the animal model of HT, it was observed that selenium supplementation increased the level of this group of lymphocytes. Moreover, the concentrations of anti-thyroglobulin antibodies and lymphocytes infiltrating thyroid tissue decreased at that time [14]. Treg cells are less sensitive to oxidative stress compared to Th [15], and it may be due to the increased expression of the antioxidant protein thioredoxin 1 in these lymphocytes, the reduction of which is catalyzed by the selenoenzyme thioredoxin reductase [16]. Another example of the potential effects of selenium on the immune system, important in the context of HT, is related to the reduction of the expression of HLA-DR (human leukocyte antigen–DR isotype) antigen in thyrocytes. This effect may be explained by the antioxidant properties of selenium, as an inverse correlation was noted between total antioxidant state and the expression of HLA-DR in these cells [17]. Selenium may also inhibit thyrocyte apoptosis even in the presence of stimulating factors [18].

Taking into account the immunoregulatory properties of selenium, as well as changes in the activity of T cells potentially relevant in the pathogenesis of HT (i.e., Th1, Th2, Th17, and Treg), we attempted to assess the effect of selenium supplementation in patients with newly diagnosed disease on functions of the immune system by determining the concentrations of selected cytokines secreted by these groups of lymphocytes.

To better understand the potential mechanisms of selenium influence on the development of HT, the search for dependencies among the above-mentioned cytokines, as well as other parameters previously determined in this group of patients [19], was undertaken, using the appropriate statistical model. It is worth noting that this research problem is extremely rare in the literature on selenium supplementation in patients with HT.

## 2. Materials and Methods

### 2.1. Study Design

Detailed descriptions of the patients and study design are included in our previous article [19]. Briefly, a group of 29 women (mean: age 37.0 ± 7.4 years, BMI 23.3 ± 4.3 kg/m^2^) with newly diagnosed and previously untreated Hashimoto’s thyroiditis with euthyroid or subclinical hypothyroidism participated in the study. None of these patients had other comorbidities, and none used medications or preparations containing vitamins/minerals permanently. Furthermore, these patients did not use a special diet, and all were non-smokers.

Participants of this study received selenium at a dose of 100 µg/day as sodium selenite (IV) for a 6-month study period. Seventeen women simultaneously received L-T4, but they did not constitute a separate group in this study. Doses of the drug were set individually and ranged from 12.5–50 µg/day. Immunological markers, as well as previously described parameters: selected markers of thyroid function (thyrotropic hormone (TSH), free thyroxine (fT4), total triiodothyronine (T3), anti-thyroid peroxidase (anti-TPO)); markers of the selenium status (serum selenium (Se), selenoprotein P (SELENOP), glutathione peroxidase 3 (GPX3)); and other biochemical parameters (ferric ion reducing antioxidant power (FRAP), total cholesterol (CHOL), LDL cholesterol (LDL), HDL cholesterol (HDL), triglycerides (TG) and glucose)) were determined in serum twice, i.e., before and after selenium supplementation. Ioduria (UI) was determined at three measurement points (initial (UI1), after 3 months from the beginning (UI2), and at the end (UI3) of study) [19]. In addition, total energy intake was calculated from the daily dietary history (collected 3 times during the study).

### 2.2. Methods

The determination of immunological parameters: interferon γ (IFN-γ), tumor necrosis factor α (TNF-α), chemokine CXCL10 (CXCL10, synonym: interferon gamma-induced protein 10 (IP-10)), interleukin 4 (IL-4), interleukin 1β (IL-1β), interleukin 17 (IL-17), and transforming growth factor β (TGF-β) in serum samples was performed using enzyme immunoassays (Diaclone), respectively: Human IFN-γ ELISA KIT, Human TNF-α ELISA KIT, Human IP-10 ELISA KIT, Human IL-4 ELISA KIT, Human IL-1β ELISA KIT, Human IL-17A ELISA KIT, and Human TGF-β1 ELISA KIT. The determination of C-reactive protein (CRP) in serum samples was performed using the enzyme immunoassay, BioVendor Human hsCRP ELISA KIT. The absorbance values were read at wavelength λ = 450 nm and 22 °C using the modular multi-sensing microplate reader, Synergy 2 (BioTek Instruments, Winooski, VT, USA). Tests for all these parameters were performed according to protocols provided with kits.

### 2.3. Statistical Approach

Results for immunological parameters are shown as mean ± standard deviation, except for parameters marked by #, which are expressed as mean and confidence interval (after converting to logarithms) because of their strong skewed distribution. In order to determine the normality of distribution and homogeneity of variance of analyzed parameters, the Kolmogorov–Smirnov test was used with Lilliefors correction, Shapiro–Wilk, and Levene’s tests, respectively. The significance of differences between data before and after selenium supplementation was assessed by a parametric Student’s t-test for two dependent samples (in case of parameters with normal distribution and homogeneous variances) and non-parametric Wilcoxon test (in case of parameters not fulfilling these conditions). Significant differences were assumed at *p* < 0.05. 

**Figure 1:**
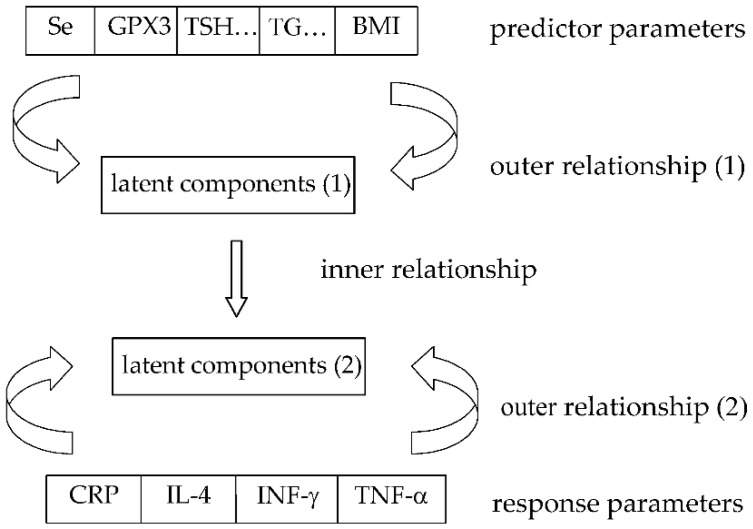
The arrow scheme for PLS algorithm. TSH… = thyroid parameters: TSH, T3, UI; TG = metabolic parameters: glucose, HDL, TG; latent components (1)—orthogonal latent scores vectors derived from selenium status, thyroid function, and metabolic parameters; latent components (2)—orthogonal latent scores vectors derived from immune system parameters.

To reveal the correlation structure between all parameters determined in the study participants (presented in this study (including immunological parameters) and in the previous work [19] (including thyroid function parameters, selenium-iodine status, and other parameters, including age, BMI, and total energy intake)), a partial least square (PLS) approach was used. The PLS model correlates the predictive parameters and response ones. The basic idea of PLS is to span the block of the original predictive parameters by mutually orthogonal latent (hidden) variables, which are linear combinations of the predictor variables. They are found by an iterative procedure to provide maximal fit to the path model, and the corresponding equation is called “outer relationship”. When there is more than one response parameter (as in our case), these response parameters can also be modeled in a similar way, and we obtain a second “outer relationship”. In the inner relationship, the latent variables from the response block are modeled by a linear combination of the latent variables from the predictor block. The final plot comprises the weights that combine the predictor parameters and response parameters with latent components, and shows, in such a way, the joint correlation structure between them. The arrow scheme for the PLS algorithm applied to our study is shown in Figure 1. In our study, the PLS model was found through testing different sets of original parameters and using a cross-validation procedure for parameter selection. Cross-validation criterion Q2 was computed as (1.0-PRESS/SS), where PRESS is the prediction error sum of squares from each prediction group, and SS is the residual sum of squares of the previous dimension; the limit for Q2 was set to be equal to 0.05. The parameters which turned out to be uninformative are discarded from the model. Uninformative means that they are irrelevant to the problem of data structure and interrelationships under investigation, i.e., they have relatively small loadings on both latent components, which means that they were not significantly correlated with other parameters. PLS was developed for analyzing complex data through reduction of their dimensionality, with a large amount of information from the original data still retained. A more detailed description of the PLS method can be found elsewhere [20,21]. A working hypothesis of our study was: in women with Hashimoto’s disease, it is possible to demonstrate, in a common statistical model, the existence of simultaneous relationships between parameters characterizing selenium status, thyroid function, immune system, and other biochemical parameters (Figure 1).

Two separate PLS models meeting the cross-validation criteria were constructed for parameters obtained before and after selenium supplementation, respectively. It was assumed that parameters with a high absolute value of weights (>0.3) along the same axis in the PLS model are correlated with each other. In order to express the strength of such correlations, the so-called correlation weights (i.e., the algebraic products of the corresponding weights and the cosine of the angle determined by the lines connecting the center of the coordinate system with the points representing these parameters in the PLS plot) were calculated for pairs of correlated parameters. Spearman’s correlation coefficients were also calculated for the same parameters to check if a correlation between these parameters could be found outside the PLS model. 

Statistical analyses were carried out using STATISTICA v. 13.3. (TIBCO Software Inc., Palo Alto, CA, USA) and SIMCA-P v. 9 (Umetrics, Umeå, Sweden) packages. The correlation weights were calculated using software delivered by MP System Co. (Chrzanów, Poland). 

## 3. Results

**Figure 2:**
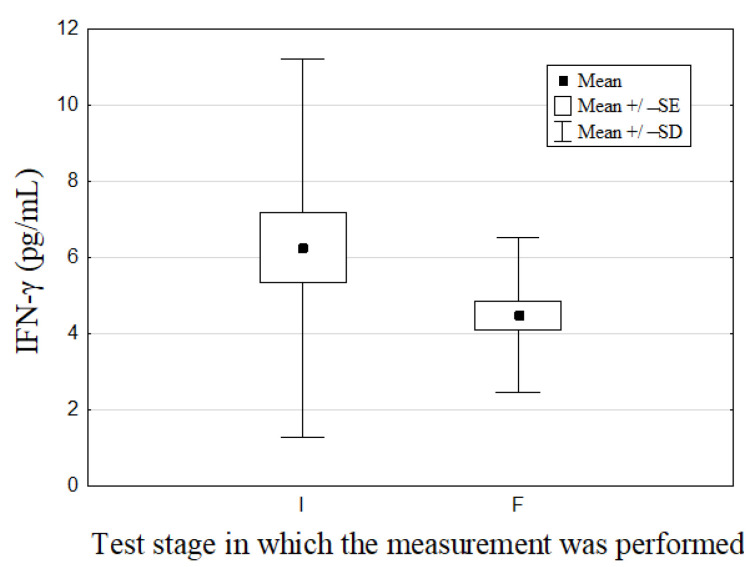
Initial (I) and final (F) values of IFN-γ in patients.

**Figure 3:**
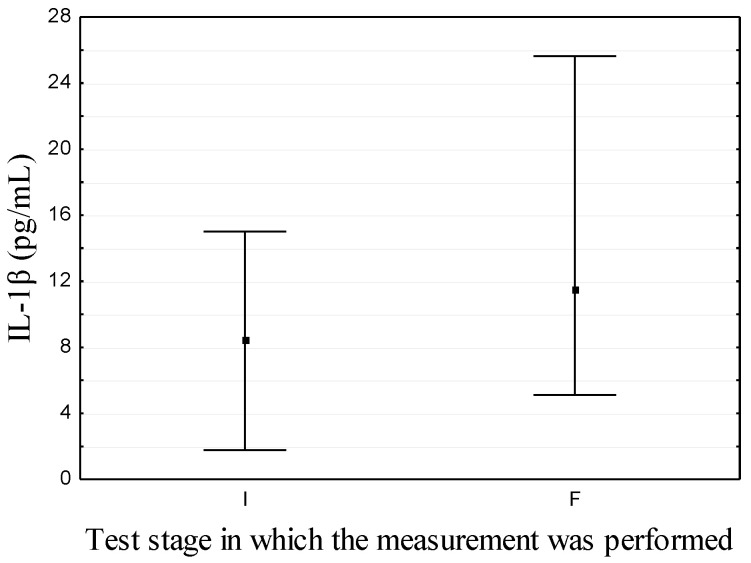
Initial (I) and final (F) values of IL-1β in patients.

Results for immunological markers are shown in Table 1 and Figure 2 and Figure 3. Among analyzed immunological parameters, significant differences were noted for the concentrations of INF-γ and IL-1β: changes from 6.25 ± 4.98 to 4.47 ± 2.02 pg/mL (*p* = 0.04) and from 8.39 ± 6.62 to 11.46 (5.12; 25.62) # pg/mL (*p* = 0.01), respectively. 

**Figure 4:**
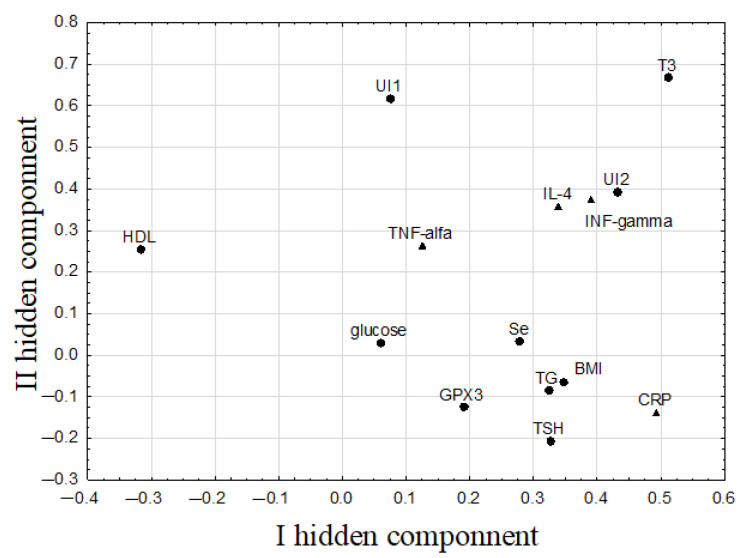
The weights of the first two hidden components in the PLS model constructed for markers of thyroid function, selenium/iodine status, and other biochemical and immunological parameters determined before selenium supplementation (dots indicate predictive parameters, triangles indicate response parameters; for simplicity, the superscript ^“I”^(initial) has been omitted from the parameter symbols).

The PLS model constructed for parameters determined before selenium supplementation (marked with superscript ^“I”^ (initial)) had three hidden components. This model explained 59.8% of variance in the prediction parameters and 55.4% of variance in the response parameters, with eigenvalues equaled to: 2.7, 1.9, and 1.4 for the first, second, and third components, respectively. The model included 10 prediction parameters (Se^I^, GPX3^I^, TSH^I^, T_3_^I^, UI^1^, UI^2^, glucose^I^, HDL^I^, TG^I^, BMI^I^) and four response parameters (CRP^I^, IL-4^I^, INF-γ^I^, TNF-α^I^), whereas others were excluded as having no informative value (Figure 4). The correlation weights of the first two hidden components calculated on the basis of the PLS model are shown in Table 2. 

The first hidden component in this model correlated most strongly and positively with T_3_^I^ and CRP^I^, as well as with UI^2^, IL-4^I^, and INF-γ^I^ (which formed a cluster of mutually correlated parameters), and negatively with HDL^I^ (Figure 4). The highest correlation weights related to this component were shown between T_3_^I^ and UI^2^, as well as T_3_^I^ and INF-γ^I^. The second component was again determined mainly by T_3_^I^, as well as UI^1^ and three other parameters with similar values of their weights in relation to this component: UI^2^, IL-4^I^, and INF-γ^I^ (Figure 4). As a result, T_3_^I^ had the highest correlation weights of all the parameters listed in Table 2. The correlations between T_3_^I^ and INF-γ^I^, as well as UI^1^ and UI^2^ were confirmed by Spearman’s rank correlation coefficients, whereas correlations between T_3_^I^ and IL-4^I^, UI^1^, and UI^2^, as well as UI^1^ and IL-4^I^ and INF-γ^I^ were revealed only by the PLS model (Table 2).

**Figure 5:**
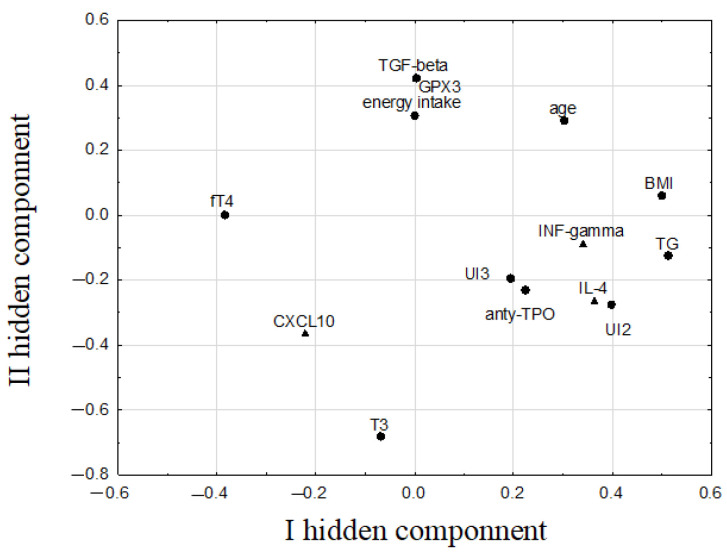
The weights of the first two hidden components in the PLS model constructed for markers of thyroid function, selenium/iodine status, and other biochemical and immunological parameters determined after selenium supplementation (dots indicate predictive parameters, triangles indicate response parameters; for simplicity, the superscript ^“F”^ (final) has been omitted from the parameter symbols).

The PLS model constructed for parameters determined after selenium supplementation (marked with superscript “^F^” (final)) also had three hidden components with eigenvalues of: 2.6, 1.7, and 1.2, respectively, which explained 55.2% and 45.1% of the variance of the prediction and response parameters, respectively. Ten prediction parameters (GPX3^F^, T_3_^F^, fT_4_^F^, anti-TPO^F^, UI^2^, UI^3^, TG^F^, BMI, age, energy intake) and four response parameters (IL-4^F^, INF-γ^F^, CXCL10^F^, TGF-β^F^) were included in this PLS model (Figure 5). The respective correlation weights are summarized in Table 3.

The first hidden component in this model correlated most strongly and positively with TG^F^ and BMI, as well as with UI^2^, IL-4^F^, and INF-γ^F^. All of these parameters had the highest positive weights related to this component; simultaneously, they correlated negatively with fT_4_^F^ (Figure 5, Table 3). The second component was determined mainly by T_3_^F^ and also by three other parameters with similar values of their weights in relation to this component: GPX3^F^, TGF-β^F^, and energy intake (Figure 5). The T_3_^F^ had negative correlation weights with these parameters (Table 3). Correlations between TG^F^ and BMI; GPX3^F^ and TGF-β^F^; BMI and INF-γ^F^; BMI and IL-4^F^; age and BMI; IL-4^F^ and INF-γ^F^; and fT_4_^F^ and INF-γ^F^ were confirmed by Spearman’s rank correlation coefficients, whereas the others listed in Table 3 were revealed only by the PLS model.

## 4. Discussion

Monitoring changes of the concentrations of anti-thyroid antibodies is one of the most frequently used elements of the methodology in research on the role of selenium in Hashimoto’s disease.

However, this is insufficient to understand the mechanisms by which this microelement is involved in regulating the production of these autoantibodies. As it was mentioned in the introduction, changes in the immune system, important in the pathogenesis of HT, are related to T lymphocytes having different functions (Th1, Th2, Th17, and Treg). Therefore, in this study, the putative selenium effect on the immune system was assessed on the basis of changes in the level of selected cytokines released by these groups of cells. In addition, we attempted to determine the correlations among these cytokines and markers of thyroid function, selenium/iodine status parameters, and other biochemical parameters (described in a previous paper), as well as age, BMI, and total energy intake (all clinical data can be found in that paper; in particular, we reported pre- and post-supplementation assessments of the patient’s selenium status, and we found statistical changes in plasma Se (75.0 ± 11.1 vs. 87.7 ± 6.3 µg/L) and selenoprotein P (SELENOP) (3.73 ± 0.49 vs. 4.40 ± 0.61 mg/L), but not in plasma glutathione peroxidase activity (GPX3; 222.9 ± 74.5 vs. 240.9 ± 118.7 U/L)) [19].

There are indications of the importance of increased Th1 lymphocyte activity in the pathogenesis of HT [5,6]. Therefore, INF-γ and TNF-α, proinflammatory cytokines secreted by these cells, were determined among the participants of the present study. six-months selenium supplementation had a significant effect on reducing the level of INF-γ in contrast to TNF-α (Table 1, Figure 2). In comparison with the results of other authors’ studies conducted in groups of healthy subjects, the results of our study were lower [22,23], higher [24], or similar [23] (Table 1). However, the presence of differences in the methodology of the cited studies (e.g., gender of participants, different immunoenzymatic kits used for cytokine determinations) should be noted, which could also affect the final results. In the course of HT, the process of thyroid tissue damage consists of several factors, e.g., increased expression of apoptotic Fas/FasL proteins and decreased expression of Bcl2 protein, an inhibitor of apoptosis within the thyrocytes [34,35]. Importantly, proinflammatory cytokines such as INF-γ can further sensitize thyrocytes to Fas/FasL-mediated apoptosis [36], as well as increase oxidative stress in them, which is another important injury factor [37]. Thus, it can be speculated that the noted change for INF-γ may be beneficial to the patient. Karanikas et al. found no significant impact of selenium supplementation (as sodium selenite (IV) at a dose of 200 μg Se/day for 3 months) on the intracellular production of INF-γ and TNF-α in CD4+ and CD8+ T cells in a group of patients with HT (N = 18) [38]. Using other methodology, Krysiak and Okopien obtained different results in their study. These authors assessed the effect of selenium supplementation (as selenomethionine at a dose of 200 μg Se/day for 6 months) on the production of INF-γ and TNF-α by lymphocytes isolated ex vivo from mononuclear cells in the peripheral blood of patients with newly diagnosed HT with euthyroidism (N = 42). The initial levels of these cytokines were: 132.5 ng/mL for INF-γ and 792 pg/mL for TNF-α. Selenium significantly decreased the concentration of these cytokines by 43% and 38%, respectively [39]

Trying to interpret the discrepancies in the results in the above-mentioned and our studies, attention should be paid to the apparent differences in the methodology of the determination of these cytokines, which make their direct comparison difficult. In light of Pilli et al.’s [40] results, it may be assumed that the impact of selenium on the immune system, including Th_1_-dependent immune response, is complex. In those studies, 6-months selenium supplementation (as selenomethionine), both at a dose of 80 μg/day and 160 μg/day, significantly reduced the levels of INF-γ and TNF-α in the serum of patients with HT with euthyroidism. The median concentrations of INF-γ and TNF-α in the group of patients with a lower dose were, respectively: 7.3 pg/mL (initial value: 9.7 pg/mL) and 9.9 pg/mL (initial value: 12.4 pg/mL), whereas with a higher dose: 7.8 pg/mL (initial value: 8.9 pg/mL) and 10.1 pg/mL (initial value: 12.1 pg/mL). However, after the next 6 months of taking selenium, these parameters again fluctuated around initial values [40].

In our study, the concentrations of chemokines, including CXCL10, were also determined. A high blood level of CXCL10 is a marker of a mainly Th_1_-dependent immune response [41,42]. There was no significant effect of selenium supplementation on the production of CXCL10 (Table 1). Esposito et al. also did not find any significant effect of selenium supplementation on this parameter in patients with newly diagnosed HT with euthyroidism (114 ± 9.7 vs. 102 ± 8.6 pg/mL) (the values read from the graph) [43]. These observations are in contrast to the above-mentioned studies of Pilli et al., who found that daily selenium supplementation for a year significantly decreased the levels of CXCL10 both at a dose of 80 μg/day and 160 μg/day, respectively: from 122.5 (50–181.1) to 93.8 (42.6–132) pg/mL, and from 141.7 (74.6–195.9) to 99.6 (85.2–149.2) pg/mL (median (min–max) - the values read from the graph). The significant effects were noted after 6 months in both groups [40]. However, it should be noted that these studies included patients with HT who had previously been diagnosed and treated pharmacologically, which constitutes an essential difference to our research.

Among cytokines secreted by Th_2_ lymphocytes, we determined IL-4, which stimulates humoral response and also inhibits the production of pro-inflammatory Th_1_-dependent cytokines, e.g., INF-γ [42,44,45]. However, in our study, 6-months selenium supplementation had no significant effect on the concentration of IL-4 in the serum of patients (Table 1). Karanikas et al. also found no significant effect of taking selenium on the change in the percentage of IL-4 producing T lymphocytes [38], despite the fact that their studies included patients previously diagnosed and treated pharmacologically. Moreover, Guclu et al. observed that restoration of euthyroidism (by L-T_4_ therapy) in HT patients with previous hypothyroidism had no significant effect on concentration of IL-4 in serum (1.44 ± 0.63 vs. 1.57 ± 0.89 pg/mL) [46].

Interestingly, the analysis of correlations among the tested parameters revealed a positive correlation between IL-4 and INF-γ, both before (Table 2) and after (Table 3) selenium supplementation, despite a significant decrease in the concentration of INF-γ at the end of study (Table 1). In light of the above results, there is a supposition that changes in Th_2_ cells activity in HT may manifest in the disturbed secretion of cytokines other than IL-4. However, the levels of this interleukin in our study were higher than those obtained in the group of healthy people in Poland by Zajkowska et al. [27] (1.11 ± 1.07^I^; 1.07 ± 0.97^F^ vs. 0.55 ± 0.24 pg/mL, Table 1). Both IL-4 and INF-γ correlated with thyroid hormone levels and ioduria. IL-4^I^ and INF-γ^I^ positively correlated with T_3_^I^, UI^1^, and UI^2^ (Table 2); in turn, IL-4^F^ and INF-γ^F^ positively correlated with UI^2^ and negatively with fT_4_^F^ (Table 3). These correlations indicate the complexity of interactions of the particular subpopulations of T lymphocytes (mediated by produced cytokines), and also the possibility of their modification depending on the functional state of the thyroid gland (i.e., hormone production) and the iodine supply with diet, which is consistent with the results of other authors [9,47]. Nevertheless, this issue requires further research. The lack of ioduria at the third measurement point (UI^3^) in the above correlations is puzzling. However, its value in combination with a negative correlation between fT_4_^F^ and UI^2^ (Table 3) enables a hypothesis to be drawn that it was a predictor of decreased iodine status in participants. 

Other parameters determined in this study were IL-1β [48,49,50] and IL-17 [42,51,52], which are associated with the pathogenesis of HT. In the study of Phenekos et al., the level of IL-1β in the serum of patients with HT at different stages of disease (TSH > 8 μIU/mL; some participants were treated with L-T_4_ for up to 18 months) was significantly lower than in the group of healthy subjects (2.52 ± 0.14 vs. 3.6 ± 0.20 pg/mL) [29]. In our study, after 6-months selenium supplementation, the level of IL-1β increased significantly (Table 1, Figure 3). Both levels (initial and final) were higher in comparison with the results of studies conducted in groups of healthy subjects [28,29] (Table 1). Moreover, taking into account the potential role of this cytokine in the process of thyroid tissue damage in HT (similarly to INF-γ, as mentioned above) [36,48,53], as well as the stimulation of virgin T lymphocytes to differentiate into Th17 cells and their secretion of IL-17, which has also been associated with the pathogenesis of this disease [42,51,52], the observed change is not beneficial to the patient. Thus, selenium may not be effective in inhibiting unfavorable changes in the production of this cytokine. It is likely that the stage of disease development and degree of thyroid dysfunction are important in shaping the profile of secreted cytokines (in this case, IL-1β) by cells participating in the autoimmunity process. The premise for such a conclusion is the results of Altay et al., who found significant differences in the level of IL-17 between group of patients with newly diagnosed HT with euthyroidism (median (min–max): 2.0 (1.1–2.7) pg/mL) and those with subclinical hypothyroidism (median (min–max): 2.3 (1.6–10.7) pg/mL) or overt hypothyroidism (median (min–max): 2.4 (1.5–7.8) pg/mL) [30]. In our study, there was no significant effect of selenium on the production of IL-17 (Table 1). Nevertheless, the lack of studies conducted in a European population of patients with HT supplemented with selenium makes the interpretation of these results difficult. It is worth noting that the values of this cytokine observed in this study, both before and after supplementation, were higher compared to the results of other studies conducted in groups of healthy people in Poland (2.5 ± 1.11 pg/mL [54]; 3.47 ± 2.65 pg/mL [27]).

As mentioned in the introduction, the role of excessive activation of Th_1_ and Th_17_ in the development of immunological disorders accompanying HT seems to be an important issue. In light of the study of Li et al., processes leading to the dominance of one of these cell groups may be very complex, as environmental factors such as iodine supply may shape them in parallel to the stage of disease. High iodine supply promoted the differentiation of naive T cells into Th1, and lower iodine supply into Th17 [47]. These observations may provide a partial explanation for some of the correlations revealed in our study, i.e., positive correlation between INF-γ (Th_1_-dependent cytokine) with ioduria (INF-γ^I^ with UI^1^ and UI^2^ (Table 2) and INF-γ^F^ with UI^2^ (Table 3)), and a lack of correlation between IL-17 (Th_17_-dependent cytokine) and urinary iodine. The median ioduria over 6 months of our study [19] was within the range that indicated normal iodine supply [55]; however, in comparison with Zagrodzki et al.’s study [56], it may indicate an increasing iodine intake in Poland. Thus, it is possible that in the process of the iodine modulation of the differentiation of naive T lymphocytes, not only is the amount of iodine supplied to the body important, but so is the general trend of its consumption in a given period. Considering the significant effect of selenium supplementation on a decreasing INF-γ level noted in our study (Table 1), it may be plausible that selenium partially eliminates the immunomodulatory properties of iodine in HT patients with euthyroidism or subclinical hypothyroidism.

Treg cells participate in the suppression of the autoimmunity process among others by the production of TGF-β [42,57,58]. Glick et al. observed in patients with autoimmune thyroid diseases, including HT, a limited ability of these cells to inhibit the proliferation of T effector cells compared to healthy people [59]. Secondly, a reduced production of TGF-β may promote the development of autoimmune diseases [60,61]. Therefore, that study also analyzed TGF-β, especially since the assessment of the effect of selenium on the regulation of its concentration in patients with HT is a little-known issue so far. However, in our study, 6-months selenium supplementation had no significant effect on the concentration of TGF-β in the serum of patients (Table 1). On the other hand, an analysis of the relationship among parameters determined after 6-months selenium supplementation in the group of patients with HT revealed significant correlations between TGF-β^F^ and other parameters, such as GPX3^F^, CXCL10^F^, and T_3_^F^ (Table 3). TGF-β^F^ and GPX3^F^ correlated positively. Although GPX3^F^ activity was not significantly higher when compared with GPX3^I^, there was a significant increase in both Se and SELENOP levels [19] after selenium supplementation, which may indirectly demonstrate the role of selenium in suppressing TGF-β-mediated autoimmunity. Moreover, TGF-β^F^ and GPX3^F^ correlated negatively with CXCL10^F^ (Table 3)—a chemokine associated with Th_1_-dependent immune response, despite the lack of significant change in its level after Se supplementation. However, it should be added that such a change was observed for INF-γ, which promotes CXCL10 production. We also found a negative correlation between T_3_^F^ and GPX3^F^ and TGF-β^F^, as well a positive correlation with CXCL10^F^. These correlations have not previously been reported by other authors, which makes their interpretation difficult, and they obviously do not have to imply causal relationships between these parameters.

Since the anti-inflammatory protein, C-reactive protein (CRP), was usually not addressed in research on the effects of selenium on the course of HT, we also included it in the study. There was no significant effect of selenium supplementation on the concentration of CRP (Table 1). Values of this parameter (both before and after supplementation) were lower than results from another study conducted in patients with newly diagnosed and previously untreated HT in Poland [39], in which the level of CRP ranged from 8.0–8.8 mg/L, and after 6 months of selenium supplementation, decreased significantly to 4.4 ± 0.7 mg/L. One of the potential reasons for discrepancies in these results may be differences in the initial levels of anti-TPO in patients (i.e., 272 IU/mL (117.48–630.89 IU/mL) (mean and the confidence interval obtained after converting data to logarithms)) in present study vs. 1761 ± 375 IU/mL [39]; determination of CRP in different blood fractions (serum vs. plasma), as well as form and dose of selenium used for diet supplementation (sodium selenite (IV), 100 μg/day vs. selenomethionine, 200 μg/day)).

The statistical analysis among parameters determined before selenium supplementation revealed a positive correlation between CRP^I^ and INF-γ^I^ (Table 2); however, this correlation was not found at the end of the study. The value of INF-γ^F^ was significantly lower than INF-γ^I^, whereas CRP^F^ levels still oscillated around the baseline value (Table 1). The initial concentration of anti-TPO [19] was lower compared to other studies that also included patients with newly diagnosed and previously untreated Hashimoto’s disease [39,43], which may have been partly reflected in the higher levels of CRP in studies by other authors.

In studies similar to ours, Marchiori et al. also reported no significant changes in CRP concentration, despite a significant decrease in INF-γ and TSH, and an increase in fT_4_ levels, after one year of L-T_4_ therapy in a group of patients with newly diagnosed hypothyroidism due to HT. Their patients’ initial anti-TPO levels were close to those in our study (398 ± 310 IU/mL [62] vs. 272 IU/mL (117.48–630.89 IU/mL)^2^). It is also possible that CRP is not an optimal marker of low-grade inflammation, as shown by studies of Erden et al. [63].

CRP is an independent factor that increases cardiovascular risk [64], and has been associated with lipid metabolism [65,66]. CRP^I^ positively correlated with BMI, TG^I^, and UI^2^, and negatively with HDL^I^. In addition, a positive correlation of CRP^I^ was also reported in relation to TSH^I^ and T_3_^I^ (Table 2). Roef et al., in a population-based study involving euthyroid subjects, reported a positive correlation between CRP and T_3_ and TSH, as well as a negative correlation with fT_4_ [67].

In the case of other dependencies not related to immunological parameters, the PLS model revealed a positive correlation of ioduria at the second measurement point (UI^2^) with BMI (Table 2). This correlation, combined with a negative correlation of fT_4_^F^ with BMI and T_3_^F^ with total energy intake (Table 3), may indicate an effect of the caloric composition of the diet on the rate of thyroid hormone deiodination [68,69,70], but this hypothesis requires further research in order to verify it. The positive correlations between T_3_^I^ and UI^1^ and UI^2^ (Table 2) confirm a link between thyroid hormone synthesis and iodine metabolism in the body. Triiodothyronine present in the bloodstream is primarily derived from T_4_ conversion, in which selenoproteins from the iodothyronine deiodinases (DIO) family are involved [71]. It can be speculated that under conditions of increased selenium supply, DIO activity increased among the participants of this study, accelerating the rate of the above-mentioned conversion. Such a phenomenon gives a potential explanation for the negative correlation between fT_4_^F^ and UI^2^ (Table 3). The lack of such correlations with ioduria determined at the end of the study (UI^3^) may indicate fluctuations in the supply of this element in the diet of patients.

The levels of thyroid hormones (fT_4_ and T_3_) were within the range of normal values (at each stage of our study) [19]. Similarly, the mean value BMI of participants indicated normal body weight, although dietary energy intake (1794.9 ± 404.1 kcal/day) was below the mean norm for individual women (2331.0 kcal/day) (average of normal values for individual participants in our study). Therefore, it is possible that even small changes in thyroid function, as manifested by changes in thyroid hormone levels within the normal range, may have affected BMI through small, but long-term, changes in the body’s energy metabolism, consistent with the assumption made by Knudsen et al. [72]. In addition, a positive correlation between TSH^I^ and BMI was noted (Table 2), which is consistent with the results of other studies conducted among people without thyroid dysfunction [73,74,75,76], as well as studies involving children with primary hypothyroidism treated for at least 6 months with L-T_4_ to restore euthyroidism [77]. Siemińska et al. also noted a dependence between TSH and BMI among postmenopausal women both with properly functioning thyroid and subclinical hypothyroidism (there were cases in that study with anti-TPO antibodies present) [78]. Increased leptin concentration, disturbances in activity of DIO [69,79], as well as a decreased expression of genes encoding TSH receptors [80] may potentially explain the above-mentioned correlation.

Simultaneously, thyroid function markers (TSH^I^, T_3_^I^, fT_4_^F^) correlated with lipid parameters (Table 2 and Table 3), thus confirming the influence of the thyroid on lipid metabolism. Wolffenbuttel et al., in a population-based study, reported correlations between parameters characterizing thyroid function and selected lipid markers, including negative correlations between fT_3_ and HDL, and between fT_4_ and TG, and a positive correlation between TSH and TG [70]. The correlations between thyroid physiological status and selected lipid parameters have also been observed by other authors [67,81,82,83]. The participants in the aforementioned studies were euthyroid.

The correlations listed in Table 2 and Table 3, which have not been discussed, have not previously been described in the literature consistent with the subject of our study, and therefore, explaining their importance at this stage of scientific development is difficult.

There are some limitations of our study. First, there is a lack of control group. However, at this stage of the study, the most important thing was to track changes in immunological parameters and other biochemical parameters in the same group of patients with Hashimoto’s disease who constitute the control group for themselves. This research was conducted according to a within-subjects design, where all participants studied were exposed to the same treatment. On the other hand, it was proved that healthy adults show very stable immune cell frequencies and serum protein levels over time. Thus, it can be assumed that the values of the analyzed immunological parameters, which can be different in different populations (Table 1), can actually remain stable in healthy individuals [84,85]. In our study, some immunological parameters were determined for the first time in such a group of patients receiving selenium. Nobody has studied the relationships between them before. The restriction at the recruitment stage allowed us to gather a study group that was relatively homogeneous in terms of disease severity, and also strongly influence the dynamics of the inclusion of individual participants in our study. 

Second, this study had a relatively small number of participants, and a lack of monitoring thyroid gland status through power Doppler sonography. The inclusion and correcting of all these factors and circumstances in further research should define more precisely the clinical relevance of selenium supplementation and the possible mechanisms of its action in such patients.

Overall, our study aimed to better understand the mechanisms by which selenium may influence the production of anti-thyroid autoantibodies in HT by determining changes in the concentration of selected cytokines in the serum of patients with newly diagnosed and previously untreated HT, with euthyroidism or subclinical hypothyroidism, receiving sodium selenite (IV) as a diet supplement. The concentration of INF-γ significantly decreased and the concentration of IL-1β significantly increased upon selenium supplementation. The other immunological parameters analyzed were apparently independent of selenium supplementation. In our study, we used an inorganic form of selenium to supplement the diet of patients. The rationale for the use of such a form is that inorganic selenium compounds are more reactive and are metabolized faster than organic ones. Therefore, selenium in such compounds may have a stronger influence on the immune system.

The scope of the analyzed parameters is undoubtedly the strength of this study. However, based on the analysis of the identified correlations and the results of other authors, the question may still be asked: to what extent do environmental factors, such as caloric content/diet composition (including selenium and iodine supply), influence the induction and severity of inflammation in the early stages of HT, and to what extent is it influenced by endogenous factors, such as the physiological state of the thyroid gland, especially with relatively low levels of anti-TPO antibodies?

## 5. Conclusions

The stage of HT development, degree of thyroid dysfunction, and selenium supplementation of the diet are interdependent factors which shape the profile of some cytokines secreted by cells participating in the autoimmunity process. It is possible that even slight fluctuations in the thyroid hormone levels in the normal range partially translate into changes in selected cytokines, CRP, and lipid parameters. Therefore, it is necessary to continue this aspect of research in order to determine the significance of the observed changes in cytokine production, and to better understand the influence of selenium on the immune system in the course of HT.

## Figures and Tables

**Table 1:** The levels of immunological parameters in serum of patients (*n* = 29) with Hashimoto’s thyroiditis with euthyroidism or subclinical hypothyroidism before and after 6-months selenium supplementation (IFN-γ—interferon γ; TNF-α—tumor necrosis factor α; CXCL10—chemokine CXCL10; IL-4—interleukin 4; IL-1β—interleukin 1β; IL-17—interleukin 17; TGF-β—transforming growth factor β; CRP—C-reactive protein).

Parameters	Initial Values	Final Values	*p* Value	Normal Values ^
INF-γ (pg/mL)	6.25 ± 4.98	4.47 ± 2.02	*p* = 0.04	(7.0 ± 3.9) ^a^–(15.0 ± 4.0) ^a^ [22,23]
TNF-α (pg/mL)	10.66 (2.83; 40.24) #	9.69 (2.99; 31.33) #	*p* > 0.05	(1.25 ± 2.20) ^a^–(12.0 ± 4.0) ^a^ [23,24]
CXCL10 (pg/mL)	179.7 ± 104.0	191.8 ± 153.8	*p* > 0.05	(25.9 (7.9–95.9)) ^b^–(88.83 ± 28.68) ^a^ [25,26]
IL-4 (pg/mL)	1.11 ± 1.07	1.07 ± 0.97	*p* > 0.05	(0.55 ± 0.24) ^a^ [27]
IL-1β (pg/mL)	8.39 ± 6.62	11.46 (5.12; 25.62) #	*p* = 0.01	(1.48 ± 0.70) *^,^^a^–(3.60 ± 0.20)^a^ [28,29]
IL-17 (pg/mL)	11.31 (3.60; 35.54) #	8.00 (2.35; 27.23) #	*p* > 0.05	(1.8 (1.2–2.3)) ^b^–(4.04 (4.17)) ^c^ [9,30]
TGF-β (ng/mL)	7.33 ± 2.60	7.12 ± 2.08	*p* > 0.05	(16.31 ± 3.21) ^a^ [31]
CRP (mg/L)	0.23 (0.03; 1.87) #	0.21 (0.03; 1.53) #	*p* > 0.05	(0.15 ± 0.13) ^a^–(2.6 ± 4.0) ^a^ [32,33]

# mean and the confidence interval obtained after converting data to logarithms. ^ ranges of values for groups of healthy people reported by other authors. * group of children. ^a^ value indicated as mean ± standard deviation. ^b^ value indicated as median (range). ^c^ value indicated as median (interquartile range).

**Table 2:** The correlation weights based on the PLS model constructed for markers of thyroid function, selenium/iodine status, and other biochemical and immunological parameters determined before selenium supplementation (twenty-five pairs of correlated parameters with highest absolute values of correlation weights are given; for simplicity, the superscript ^“I”^(initial) was omitted in the parameter symbols).

Pairs of Correlated Parameters		Correlation Weights	Spearman’s Rank Correlation Coefficients and Significance Level
T_3_	UI^1^	0.355	(N.S.)
T_3_	UI^2^	0.259	(N.S.)
T_3_	INF-γ	0.248	0.537 (*p* = 0.003)
T_3_	IL-4	0.239	(N.S.)
UI^1^	UI^2^	0.184	0.520 (*p* = 0.004)
UI^1^	INF-γ	0.179	(N.S.)
UI^1^	IL-4	0.178	(N.S.)
BMI	CRP	0.169	0.500 (*p* = 0.006)
UI^2^	INF-γ	0.169	(N.S.)
TG	CRP	0.158	(N.S.)
TSH	CRP	0.153	(N.S.)
UI^2^	IL-4	0.146	(N.S.)
IL-4	INF-γ	0.134	0.624 (*p* = 0.000)
UI^2^	CRP	0.113	(N.S.)
TG	BMI	0.111	(N.S.)
TSH	BMI	0.104	(N.S.)
TSH	TG	0.100	0.383 (*p* = 0.040)
INF-γ	CRP	0.098	0.465 (*p* = 0.011)
T_3_	CRP	0.094	(N.S.)
UI^2^	BMI	0.090	(N.S.)
HDL	CRP	−0.091	−0.394 (*p* = 0.034)
HDL	IL-4	−0.107	(N.S.)
HDL	INF-γ	−0.124	−0.445 (*p* = 0.016)
HDL	UI^2^	−0.137	(N.S.)
T_3_	HDL	−0.158	−0.397 (*p* = 0.033)

N.S.: statistically nonsignificant.

**Table 3:** The correlation weights based on the PLS model constructed for markers of thyroid function, selenium/iodine status, and other biochemical and immunological parameters determined after selenium supplementation (twenty-five pairs of correlated parameters with highest absolute values of correlation weights are given; for simplicity, the superscript ^“F”^(final) was omitted in the parameter symbols).

Pairs of Correlated Parameters		Correlation Weights	Spearman’s Rank Correlation Coefficients and Significance Level
TG	BMI	0.239	0.436 (*p* = 0.018)
T_3_	CXCL10	0.224	(N.S.)
TG	UI^2^	0.190	(N.S.)
GPX3	TGF-β	0.181	0.632 (*p* = 0.000)
TG	INF-γ	0.175	(N.S.)
TG	IL-4	0.173	(N.S.)
BMI	INF-γ	0.158	0.373 (*p* = 0.046)
UI^2^	BMI	0.148	(N.S.)
UI^2^	IL-4	0.144	(N.S.)
BMI	IL-4	0.133	0.379 (*p* = 0.042)
GPX3	energy intake	0.132	(N.S.)
energy intake	TGF-β	0.132	(N.S.)
UI^2^	INF-γ	0.127	(N.S.)
age	BMI	0.120	0.601 (*p* = 0.001)
IL-4	INF-γ	0.115	0.578 (*p* = 0.001)
fT_4_	IL-4	−0.113	(N.S.)
fT_4_	UI^2^	−0.125	(N.S.)
fT_4_	INF-γ	−0.128	−0.447 (*p* = 0.015)
CXCL10	TGF-β	−0.132	(N.S.)
GPX3	CXCL10	−0.132	(N.S.)
fT_4_	BMI	−0.192	(N.S.)
fT_4_	TG	−0.192	(N.S.)
T_3_	energy intake	−0.210	(N.S.)
T_3_	TGF-β	−0.288	(N.S.)
T_3_	GPX3	−0.288	(N.S.)

N.S.: statistically nonsignificant.

## Data Availability

The data presented in this study are available upon reasonable request from the corresponding author. The data are not publicly available due to data safety reasons.
